# Mitochondria-Derived Reactive Oxygen Species Play an Important Role in Doxorubicin-Induced Platelet Apoptosis

**DOI:** 10.3390/ijms160511087

**Published:** 2015-05-15

**Authors:** Zhicheng Wang, Jie Wang, Rufeng Xie, Ruilai Liu, Yuan Lu

**Affiliations:** 1 Department of Laboratory Medicine, Huashan Hospital, Shanghai Medical College, Fudan University, Shanghai 200040, China; E-Mails: Jiewang2015@126.com (J.W.); liuruilai88@163.com (R.L.); 2 Blood Engineering Laboratory, Shanghai Blood Center, Shanghai 200051, China; E-Mail: xierufeng555@163.com

**Keywords:** platelets, mitochondria, reactive oxygen species, doxorubicin, apoptosis

## Abstract

Doxorubicin (DOX) is an effective chemotherapeutic agent; however; its use is limited by some side effects; such as cardiotoxicity and thrombocytopenia. DOX-induced cardiotoxicity has been intensively investigated; however; DOX-induced thrombocytopenia has not been clearly elucidated. Here we show that DOX-induced mitochondria-mediated intrinsic apoptosis and glycoprotein (GP)Ibα shedding in platelets. DOX did not induce platelet activation; whereas; DOX obviously reduced adenosine diphosphate (ADP)- and thrombin-induced platelet aggregation; and impaired platelet adhesion on the von Willebrand factor (vWF) surface. In addition; we also show that DOX induced intracellular reactive oxygen species (ROS) production and mitochondrial ROS generation in a dose-dependent manner. The mitochondria-targeted ROS scavenger Mito-TEMPO blocked intracellular ROS and mitochondrial ROS generation. Furthermore; Mito-TEMPO reduced DOX-induced platelet apoptosis and GPIbα shedding. These data indicate that DOX induces platelet apoptosis; and impairs platelet function. Mitochondrial ROS play a pivotal role in DOX-induced platelet apoptosis and GPIbα shedding. Therefore; DOX-induced platelet apoptosis might contribute to DOX-triggered thrombocytopenia; and mitochondria-targeted ROS scavenger would have potential clinical utility in platelet-associated disorders involving mitochondrial oxidative damage.

## 1. Introduction

Doxorubicin (DOX) has been used for the treatment of solid tumors and hematologic malignancy. However, DOX therapy has some side effects, such as thrombocytopenia [1,2]. Up to now, the pathogenesis of DOX-induced thrombocytopenia is not completely understood. Recently, several studies have reported that DOX can induce platelet cytotoxicity and procoagulant activity [2,3]. It has been generally accepted the anticancer effects of DOX via inducing apoptosis of malignant cell [4,5]. Platelet apoptosis induced by either physiological or chemical compounds occurs widely *in vitro* or *in vivo* [6,7,8,9,10], which might play important roles in controlling the number of circulating platelets or in the development of platelet-related diseases. Accumulating evidences indicate that platelet apoptosis might play a key role in chemotherapeutic agents induced-thrombocytopenia [8,9].

DOX localizes to the mitochondria and is highly susceptible to enzymatic reduction to generate ROS, which can cause mitochondrial swelling and ultrastructural changes and alter mitochondrial function [11]. Recently, most studies supported that the major mechanism of DOX-induced apoptosis was related to excessive generation of intracellular ROS [11,12,13,14,15,16]. Mitochondria are considered the main intracellular source of ROS [17]. ROS are produced at very low levels during mitochondrial respiration under normal physiological conditions. The formation of ROS occurs when unpaired electrons escape the electron transport chain and react with molecular oxygen, generating ROS. Complexes I, II, and III of the electron transport chain are the major potential loci for ROS generation [18,19]. Recently, several studies reported that NADPH oxidase 4 (NOX4) localizes to mitochondria, and NOX4 is a novel source of ROS produced in the mitochondria [20,21]. ROS degradation is performed by endogenous enzymatic antioxidants, such as superoxide dismutase, catalase, and non-enzymatic antioxidants, such as glutathione, ascorbic acid [17]. Under physiological conditions, ROS are maintained at proper levels by a balance between its synthesis and its elimination. An increase in ROS generation, a decrease in antioxidant capacity, or a combination both will lead to oxidative stress [17].

In recent years, mitochondria-targeted ROS antagonists and mitochondrial ROS detection probes have been developed. Thus, with the advent of such tools, the importance of mitochondrial ROS in cell signaling, proliferation and apoptosis gradually attracted much attention. For example, Cheung *et al.* [12] recently reported that SIRT3 prevents DOX-induced mitochondrial ROS production in H9c2 cardiomyocytes. Increased mitochondrial ROS is a significant contributor to the development of DOX-induced myopathy in both cardiac and skeletal muscle fibers [13].

We recently reported that mitochondrial ROS play important roles in hyperthermia-induced platelets [7]. In the present study, using mitochondria-targeted ROS scavenger and mitochondrial ROS detection probe, we explored whether DOX induces mitochondrial ROS production, and whether mitochondria-targeted ROS scavenger has a protective effect on DOX-induced platelet apoptosis.

## 2. Results

### 2.1. Doxorubicin (DOX) Dose-Dependently Induces ΔΨm Depolarization and Phosphatidylserine (PS) Exposure in Platelets

**Figure 1:**
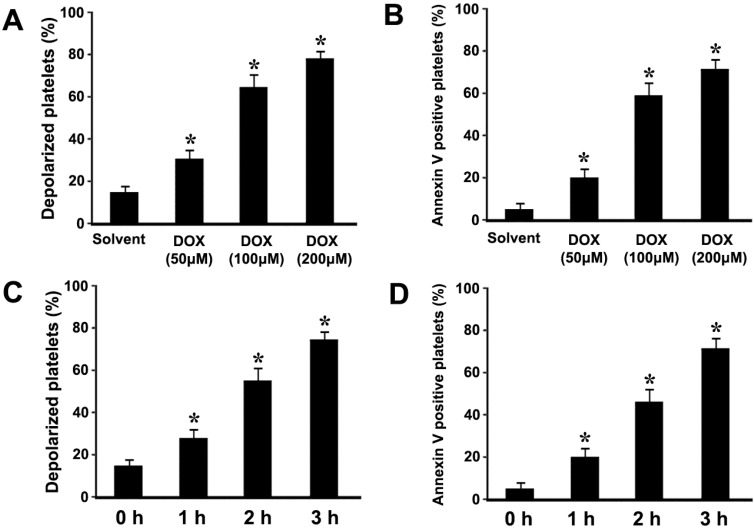
Doxorubicin (DOX) induced mitochondrial inner transmembrane potential (ΔΨm) depolarization and PS exposure. (**A**–**D**) Platelets were incubated with different concentrations of DOX or solvent control (**A**,**B**), or incubated with DOX (200 μM) at 37 °C for different times (**C**,**D**). Treated platelets were incubated with tetramethylrhodamine ethyl ester (TMRE) (**A**,**C**), or fluorescein isothiocyanate (FITC)-conjugated annexin V (**B**,**D**), and analyzed by flow cytometry. ΔΨm depolarization was quantified as the percentage of depolarized platelets. Means ± SEM from three independent experiments are shown (**A**,**C**). PS exposure was quantified as the percentage of PS positive platelets. Means ± SEM of the percentage of PS positive platelets from three independent experiments are shown (**B**,**D**). * *p* < 0.017 (after a Bonferroni correction) compared with solvent control.

In order to investigate whether DOX could induce platelet apoptosis, platelets were incubated with different concentrations of DOX. The effect of DOX on platelet ΔΨm depolarization and PS exposure is analyzed by flow cytometry. We found that DOX dose-dependently induced ΔΨm depolarization and PS exposure (Figure 1A,B). In order to investigate the effect of incubation time on apoptosis, platelets were incubated with DOX for different times. The data indicate that DOX time-dependently induced ΔΨm depolarization and PS exposure (Figure 1C,D). Therefore, in order to obtain obvious apoptotic events, 3 h incubation was selected for the following experiments.

### 2.2. DOX Dose-Dependently Induces Mitochondrial Translocation of Bax, Cytochrome C Release, and Caspase-3 Activation in Platelets

**Figure 2:**
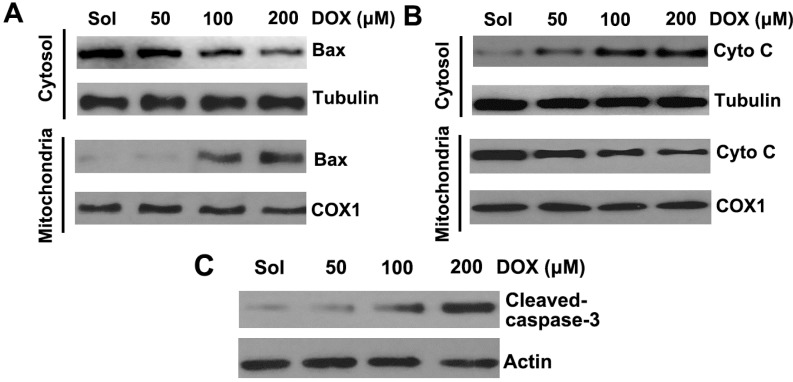
DOX induced mitochondrial translocation of Bax, cytochrome C release, and caspase-3 activation. (**A**,**B**) Platelets were incubated with different concentrations of DOX or solvent. Treated platelets were lysed, and cytosol and mitochondrial fractions were isolated and analyzed by Western blot with anti-Bax (**A**), and anti-cytochrome C antibodies (**B**), COX1 and tubulin were used as internal controls; (**C**) Platelets were incubated with different concentrations of DOX or solvent. Treated platelets were lysed and analyzed by Western blot with anti-caspase-3 antibody. Actin levels were assayed to demonstrate equal protein loading. Representative data of three independent experiments are presented. Cytochrome C is labeled as Cyto C.

Pro-apoptotic protein Bax translocation to the mitochondria is a key event that regulates the release of apoptogenic factors like cytochrome C from the mitochondria, which leads to activation of caspases such as executioner caspase-3 [22]. Thus, to further explore whether DOX could induce mitochondrial translocation of Bax and cytochrome C release, platelets were incubated with different concentrations of DOX and subjected to isolation and analysis of cytosolic and mitochondrial fractions. We found that DOX dose-dependently promoted mitochondrial translocation of Bax and cytochrome C release (Figure 2A,B). Meanwhile, caspase-3 activation was examined in DOX-treated platelets. Compared with the control, the 17-kDa caspase-3 fragment, which indicated the activation of caspase-3, dose-dependently increased in platelets treated with DOX (Figure 2C). Taken together, these data suggested that DOX induced apoptotic cascades leading to platelet apoptosis.

### 2.3. DOX Impairs Platelet Function

**Figure 3:**
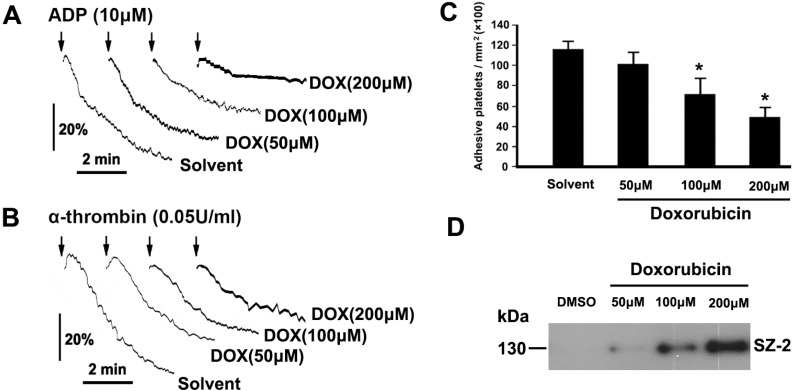
DOX impaired platelet function. (**A**,**B**) PRP or washed platelets were incubated with different concentrations of DOX or solvent. Platelet aggregation was induced by addition of ADP (**A**) or thrombin (**B**); representative traces from three independent experiments are shown; (**C**) Platelets were incubated with different concentrations of DOX or solvent. Treated platelets were perfused into vWF-coated glass capillary. The results from three independent experiments are shown as the means ± SEM of cell number/mm^2^. * *p* < 0.017 (after Bonferroni correction) as compared with solvent; (**D**) Platelets were incubated with different concentrations of DOX or solvent. Treated platelets were centrifuged, and the supernatants were analyzed by Western blot with SZ-2. Representative data of three independent experiments are presented.

Platelets play a central role in maintaining integrity of endothelium and biological hemostasis. To investigate the effect of DOX on platelet function, platelets were treated with different concentrations of DOX or solvent control, and then examined for platelet aggregation and adhesion. ADP- and thrombin-induced platelet aggregations were reduced in DOX-treated platelets in a dose-dependent manner (Figure 3A,B). Furthermore, compared with solvent control, DOX-treated platelets displayed a significant decrease in adhering on the vWF surface in dose-dependent manner (Figure 3C). Taken together, these data indicate that platelet functions are impaired by DOX.

The interaction of GPIbα with vWF at sites of injured blood vessel walls initiates platelet adhesion under flow conditions [23]. GPIbα shedding is a physiological regulatory mechanism leading to platelet dysfunction [23]. In order to investigate whether GPIbα shedding is involved in DOX-induced platelet dysfunction, GPIbα shedding was examined in platelets incubated with DOX. We found that glycocalicin, which is a cleaved production of GPIbα, gradually increased with increasing concentration of DOX (Figure 3D).

### 2.4. DOX Dose-Dependently Increases Intracellular ROS and Mitochondrial ROS Production in Platelets

**Figure 4:**
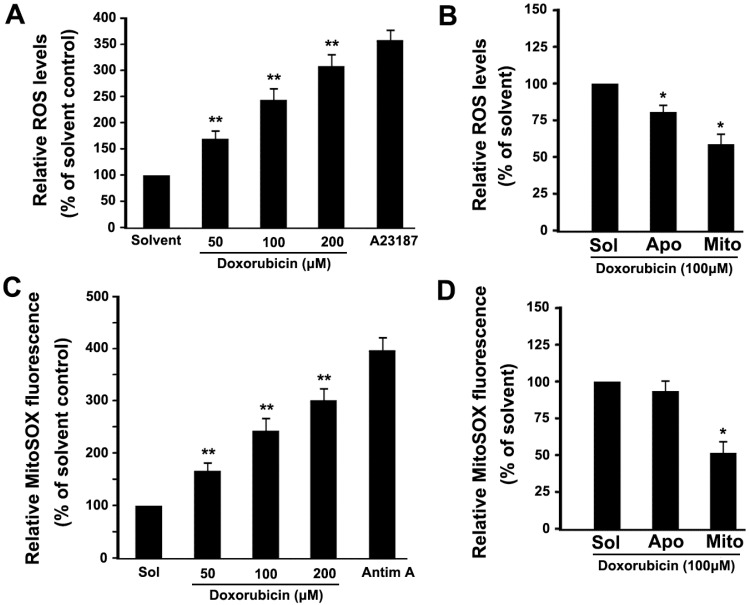
DOX increased intracellular ROS and mitochondrial ROS production. (**A**–**D**) Platelets were loaded with 2’7’-dichlorofluorescin diacetate (DCFDA) (**A**,**B**) or MitoSOX™ Red (**C**,**D**), and incubated with various concentrations of DOX or solvent (**A**,**C**), or pre-incubated with apocynin, Mito-TEMPO, and then incubated with DOX (**B**,**D**). As a positive control, loaded platelets were incubated with A23187 (3 μM) or antimycin A (50 μM) at 37 °C for 30 min. Treated platelets were analyzed for intracellular ROS or mitochondrial ROS levels by flow cytometry. The relative ROS levels are expressed as a percentage of platelets, which were incubated with solvent. Data are expressed as a percentage of platelets that were incubated with solvent control. Percentage is presented as means ± SEM from three independent experiments. ** *p* < 0.017 (after Bonferroni correction) as compared with solvent control. * *p* < 0.025 (after Bonferroni correction) as compared with solvent control. Solvent, apocynin, Mito-TEMPO and Antimycin A are labeled as Sol, Apo, Mito and Antim A, respectively.

In order to investigate whether DOX augments intracellular ROS levels in platelets, we determined platelet ROS levels using DCFDA. As shown in Figure 4A, DOX dose-dependently induced ROS production. As a positive control, A23187 significantly induces intracellular ROS production (Figure 4A). Several potential sources of ROS have been suggested, including the mitochondria and NADPH oxidase. Several reports support a role for NADPH oxidase in DOX-induced nuclear cell apoptosis [15,16]. In order to investigate the sources of ROS in DOX-treated anuclear platelets, apocynin, which is an inhibitor of NADPH oxidase, and Mito-TEMPO, which is a mitochondria-targeted ROS antagonist, were used. We found that DOX-induced ROS production was partly inhibited by apocynin, and was obviously inhibited by Mito-TEMPO (Figure 4B). These data demonstrate that mitochondria are a major source of ROS in DOX-treated platelets.

To assist in confirming that mitochondria were a major site of ROS production in DOX-treated platelets, we used MitoSOX™ Red fluorescence, which detects superoxide synthesis, to quantify mitochondrial ROS [7]. We found that DOX dose-dependently induced mitochondrial superoxide production (Figure 4C). In addition, Mito-TEMPO significantly inhibited DOX-induced mitochondrial ROS generation as compared with the solvent control (Figure 4D). Together, these observations further confirm that DOX can induce mitochondrial ROS production in platelets. As a positive control, we found that antimycin A markedly induced mitochondrial ROS production in platelets (Figure 4C).

### 2.5. DOX Dose-Dependently Increases Malonyldialdehyde (MDA) Production and Cardiolipin Peroxidation in Platelets

**Figure 5:**
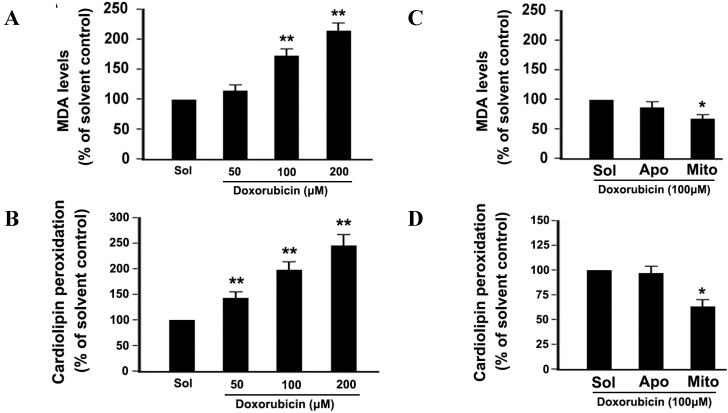
DOX increased malonyldialdehyde (MDA) production and cardiolipin peroxidation. (**A**,**B**) Platelets were incubated with different concentrations of DOX or solvent (**A**), or pre-incubated with apocynin, Mito-TEMPO, and then incubated with DOX (**B**). MDA levels were measured using an MDA assay kit. The MDA levels are expressed as a percentage of platelets that were incubated with solvent; (**C**,**D**) Platelets were incubated with different concentrations of DOX or solvent (**C**), or pre-incubated with apocynin, Mito-TEMPO, and then incubated with DOX (**D**). Cardiolipin peoxidation was detected as described in Methods. Data are expressed as a percentage of platelets that were incubated with solvent. Percentage is presented as means ± SEM from three independent experiments. ** *p* < 0.017 (after Bonferroni correction) as compared with solvent. * *p* < 0.025 (after Bonferroni correction) as compared with solvent control. Solvent, apocynin and Mito-TEMPO and are labeled as Sol, Apo and Mito, respectively.

Phospholipids are rich in unsaturated fatty acids that are particularly susceptible to ROS attack, which promotes lipid peroxidation. In order to demonstrate whether DOX treatment induces lipid peroxidation in platelets, we detected the production of MDA, which is a sensitive indicator of ROS-mediated lipid peroxidation. Production of MDA was increased in a dose-dependent manner (Figure 5A), suggesting that DOX induces platelet lipid peroxidation. Mito-TEMPO partly inhibited DOX-induced MDA production (Figure 5B). Mitochondria are the primary site of ROS generation and the major target of ROS. Cardiolipin, a unique phospholipid located at the level of the inner mitochondrial membrane, contains polyunsaturated fatty acid residues, and are thus highly prone to oxidation. In order to demonstrate whether DOX treatment induces cardiolipin peroxidation in platelets, we used the fluorescent dye NAO to estimate cardiolipin peroxidation. NAO binds to cardiolipin with high affinity, and the fluorochrome loses its affinity for peroxidized cardiolipin [7]. As shown in Figure 5C, cardiolipin peroxidation was increased in a dose-dependent manner. Mito-TEMPO obviously inhibited DOX-induced cardiolipin peroxidation (Figure 5D).

### 2.6. Mitochondrial ROS Mediates DOX-Induced Platelet Apoptosis

**Figure 6:**
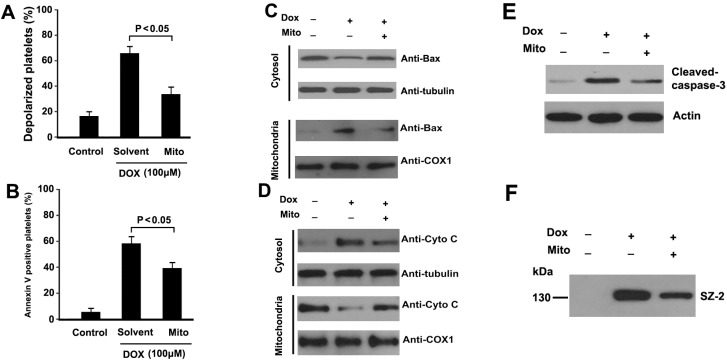
Mitochondria-targeted ROS scavenger attenuated DOX- induced platelet apoptosis. (**A**,**B**) Platelets were pre-incubated with Mito-TEMPO or solvent, and then incubated with DOX; Treated platelets were incubated with TMRE (**A**), or annexin V-FITC (**B**), and analyzed by flow cytometry. ΔΨm depolarization and PS exposure was quantified as the percentage of depolarized platelets. Means ± SEM from three independent experiments are shown; (**C**,**D**) Platelets were pre-incubated with Mito-TEMPO or solvent, and then incubated with DOX. Treated platelets were lysed, and cytosol and mitochondrial fractions were isolated and analyzed by Western blot with anti-Bax (**C**), and anti-cytochrome C antibodies (**D**); Representative results of three independent experiments are presented; (**E**) Platelets were pro-incubated with Mito-TEMPO or solvent, and then incubated with DOX. Treated platelets were lysed and analyzed by Western blot with anti-caspase-3 antibody. Actin levels were assayed to demonstrate equal protein loading. Representative results of three independent experiments are presented; (**F**) Platelets were pre-incubated with Mito-TEMPO or solvent, and then incubated with DOX. Treated platelets were centrifuged, and supernatants were analyzed by Western blot with SZ-2. Representative results of three independent experiments are presented. Mito-TEMPO is labeled as Mito.

The above observations confirmed that DOX treatment enhanced mitochondrial ROS levels in platelets. To investigate whether mitochondria-derived ROS were involved in DOX-induced platelet apoptotic events, Mito-TEMPO was pre-incubated with platelets before to DOX treatment. We found that Mito-TEMPO significantly inhibited DOX-induced platelets apoptosis, including ΔΨm dissipation, PS exposure, caspase-3 activation, mitochondrial translocation of Bax, and cytochrome C release (Figure 6A–E). Together, these data indicate that mitochondrial-derived ROS play a pivotal role in DOX-induced platelet apoptosis. In addition, Mito-TEMPO also partly inhibited DOX-induced GPIbα shedding (Figure 6F).

## 3. Discussion

DOX is a highly effective chemotherapeutic agent that is widely used to treat a variety of cancers, however, its use is limited by some side effects, such as cardiotoxicity and thrombocytopenia [1,2,11,12]. Although it has been generally accepted that DOX exerts its anticancer effect by inducing different kinds of malignant cells apoptosis, it still remains unclear whether DOX incurs platelet apoptosis. In the current observation, DOX dose-dependently induces ΔΨm depolarization, PS exposure, mitochondrial translocation of Bax, cytochrome C release and caspase-3 activation, providing sufficient evidence to indicate that DOX incurs mitochondria-mediated intrinsic platelet apoptosis. We also found that DOX did not induce platelet activation through examining P-selectin expression and PAC-1 binding (data not shown). In addition, we have tried to explore the signaling cascades leading to DOX-induced platelet apoptosis, and the data indicate mitochondrial ROS is involved in the apoptotic process.

DOX induces ROS generation and apoptosis in various cell types, and the identities of the cellular sources of ROS remain controversial. Several studies have shown that NADPH oxidase is a major source of ROS in DOX-treated cells [15,16]. In our studies, we found that NADPH inhibitor apocynin did not significantly inhibited DOX-induced platelet apoptosis (data not shown). Recently, several studies have shown that mitochondria are major source of ROS in DOX-treated cells [12,13]. We found that mitochondria are the primary source of ROS in DOX-treated platelets based on our observations that (1) the mitochondria-targeted ROS scavenger inhibited DOX-induced ROS production; and (2) DOX-induced ROS was detected by the mitochondrial ROS probe MitoSOX™ Red. Therefore, different sources of DOX-induced ROS generation are likely to be dependent on cell type.

The precise mechanisms responsible for how DOX causes increased levels of mitochondrial ROS remain undetermined. The reasons may be manifold. On the one hand, DOX might increase mitochondrial ROS production. It has been reported that DOX could induce mitochondrial dysfunction, and thus augment mitochondrial ROS generation [14]. On the other hand, DOX might provoke decreased antioxidant capacity in mitochondria. Li *et al.* reported that myocardial MnSOD mRNA was not significantly changed, but its protein levels were significantly decreased in rats treated with DOX [24].

The functional role of mitochondrial ROS in DOX-induced platelet apoptosis was determined by pre-treating platelets with a mitochondria-targeted ROS scavenger before DOX treatment and then analyzing apoptotic markers. The mitochondria-targeted ROS scavenger was found to be effective in inhibiting DOX-induced platelet apoptosis. These observations indicate that mitochondrial ROS are key mediators of DOX-induced platelet apoptosis. However, the question remains, how does mitochondrial ROS triggers platelet apoptosis? It has been previously reported that mitochondria-derived ROS plays a pivotal role in triggering apoptosis in various cell types. Several studies have shown that mitochondrial ROS easily oxidizes cardiolipin, and oxidized cardiolipin appears to be essential for mitochondrial membrane permeabilization and releases of pro-apoptotic factors into the cytosol [25]. Conversely, prevention of cardiolipin peroxidation leads to inhibition of apoptosis [26]. These findings suggest that cardiolipin might be a crucial molecule that regulates the initiation of apoptosis. Our recent data demonstrated that hyperthermia increased cardiolipin peroxidation and that mitochondrial ROS plays an important role in hyperthermia-induced cardiolipin peroxidation [7]. Future work will be necessary to define how mitochondrial ROS regulates platelet apoptosis in DOX-treated platelets.

The reasons of Dox-induced thrombocytepenia may be manifold. On the one hand, DOX might increase platelet clearance. On the other hand, DOX might inhibit megakaryocyte function and decrease platelet generation. Up to now, the effect of DOX on megakaryocyte has not been clearly elucidated. Future work will be necessary to explore how DOX influence megakaryocyte function by *in vitro* or *in vivo* experiment.

In summary, our study provides direct evidence that DOX increases mitochondria-derived ROS generation in platelets, which in turn, induces platelet apoptosis. DOX does not incur platelet activation, whereas, it impairs platelet function. These findings may reveal a mechanism for platelet clearance and dysfunction *in vivo* or *in vitro*, and also suggest a possible pathogenesis of thrombocytopenia in some patients treated with DOX.

## 4. Experimental Section

### 4.1. Reagents and Antibodies

Anti-cleaved p17 fragment of caspase-3 antibody was obtained from Millipore (Billerica, MA, USA). Mito-TEMPO was obtained from Enzo Life Sciences (Plymouth Meeting, PA, USA). A23187, DOX, adenosine diphosphate (ADP), tetramethylrhodamine ethyl ester (TMRE), apocynin, 2', and 7'-dichlorofluorescin diacetate (DCFDA) were obtained from Sigma (St. Louis, MO, USA). Monoclonal antibodies against Bax, cytochrome C, tubulin, cytochrome C oxidase subunit 1 (COX1), actin, SZ-2, and HRP-conjugated goat anti-mouse IgG were obtained from Santa Cruz Biotechnology (Santa Cruz, CA, USA). FITC-conjugated annexin V was obtained from Bender Medsystem (Vienna, Austria). MitoSOX™ Red was obtained from Invitrogen/Molecular Probes (Eugene, OR, USA). Mitochondria isolation kit was obtained from Pierce (Rockford, IL, USA).

### 4.2. Preparation of Platelet-Rich Plasma (PRP) and Washed Platelets

For studies involving human subjects, approval was obtained from the Huashan Hospital institutional review board, China. Informed consent was provided in accordance with the Declaration of Helsinki. PRP and washed platelets were prepared as described previously [7]. Briefly, fresh blood from healthy volunteers (7 males and 5 females; age range: 24–35 years) was anti-coagulated with one-seventh volume of acid-citratedextrose (ACD, 2.5% trisodium citrate, 2.0% d-glucose and 1.5% citric acid). Anti-coagulated blood was separated by centrifuging, and the supernatant was PRP. Platelets were washed twice with CGS buffer (123 mM NaCl, 33 mM d-glucose, 13 mM trisodium citrate, pH 6.5) and re-suspended in modified Tyrode’s buffer (MTB) (2.5 mM Hepes, 150 mM NaCl, 2.5 mM KCl, 12 mM NaHCO_3_, 5.5 mM d-glucose, pH 7.4) to a final concentration of 3 × 10^8^/mL, and incubated at room temperature (RT) for 1 h to recover to resting state.

### 4.3. Measurement of Mitochondrial Inner Transmembrane Potential (ΔΨm)

Washed platelets were incubated with different concentrations of DOX (50, 100, 200 μM) or solvent control at 37 °C for indicated time. TMRE was added according to a previously described method [7]. For the inhibition experiments, washed platelets were pre-incubated with Mito-TEMPO (10 μM) or solvent control at 37 °C for 15 min, and then incubated with DOX at 37 °C for 3 h.

### 4.4. Phosphatidylserine (PS) Externalization Assay

Washed platelets were incubated with different concentrations of DOX or solvent control at 37 °C for indicated time. Annexin V binding buffer was mixed according to a previously described method [7]. For the inhibition experiments, washed platelets were pre-incubated with Mito-TEMPO (10 μM) or solvent control at 37 °C for 15 min, and then incubated with DOX at 37 °C for 3 h.

### 4.5. Measurement of Intracellular ROS and Mitochondrial ROS Levels

Intracellular ROS and mitochondrial ROS levels were examined using DCFDA and MitoSOX™ Red, respectively, according to a previously described method [7]. Briefly, washed platelets were loaded with DCFDA (10 μM) or MitoSOX™ Red (5 μM) at 37 °C for 20 min in the dark and washed three times with modified Tyrode’s buffer (MTB). Pre-loaded platelets were incubated with different concentrations of DOX or solvent control at 37 °C for different time. For the inhibition experiments, pre-loaded platelets were incubated with apocynin (100 μM), Mito-TEMPO (10 μM), or solvent control at 37 °C for 15 min, and then treated with DOX at 37 °C for 3 h. A23187-treated and antimycin A-treated platelets were used as positive controls for intracellular cellular ROS and mitochondrial ROS levels, respectively.

### 4.6. Assessment of Malonyldialdehyde (MDA) Levels

Washed platelets were incubated with different concentrations of DOX or solvent control at 37 °C for 3 h. Samples were treated according to a previously described method [7]. For the inhibition experiments, washed platelets were pre-incubated with Mito-TEMPO, apocynin or solvent control at 37 °C for 15 min and then incubated with DOX at 37 °C for 3 h.

### 4.7. Assessment of Cardiolipin Peroxidation

Washed platelets were incubated with different concentrations of DOX or solvent control at 37 °C for 3 h, and then loaded with NAO according to a previously described method [7]. For the inhibition experiments, platelets were pre-incubated with Mito-TEMPO, apocynin or solvent control at 37 °C for 15 min and then incubated with DOX at 37 °C for 3 h.

### 4.8. Subcellular Fractionation

Washed platelets were incubated with different concentrations of DOX or solvent control at 37 °C for 3 h. Samples were suspended according to a previously described method [7]. For inhibition experiments, platelets were pre-incubated with Mito-TEMPO or solvent control at 37 °C for 15 min, and then further incubated with DOX at 37 °C for 3 h.

### 4.9. Western Blot Analysis

After subcellular fractionation Bax and cytochrome C were detected by Western blot using anti-Bax, and anti-cytochrome C antibodies. COX1 and tubulin were used as mitochondrial and cytosolic internal controls, respectively. Caspase-3 activation and GPIbα shedding was assessed with platelet whole lysates and supernatant, respectively. Washed platelets were incubated with different concentrations of DOX or solvent control at 37 °C for 3 h. One part treated platelets were lysed with an equal volume of lysis buffer on ice for 30 min. The samples were subjected to Western blot analysis using anti-cleaved p17 fragment of caspase-3 antibody. Anti-actin antibody was used as an equal protein loading control. Another part treated platelets were centrifuged at 4000 rpm for 5 min, and the supernatants were analyzed by Western blot with anti-GPIbα N-terminal antibody SZ-2. In the inhibition experiments, platelets were pre-incubated with Mito-TEMPO or solvent control at 37 °C for 15 min, and incubated with DOX at 37 °C for 3 h.

### 4.10. Platelet Aggregation

PRP or washed platelets were incubated with different concentrations of DOX or solvent control at 37 °C for 3 h. Platelet aggregation was induced by addition of ADP or thrombin at 37 °C with a stirring speed of 1000 rpm.

### 4.11. Platelet Adhesion under Flow Condition

The glass capillary was coated according to a previously described method [7]. Washed platelets were incubated with different concentrations of DOX or solvent control at 37 °C for 3 h, then perfused into the glass capillary by a syringe pump at a flow shear rate of 250 s^−1^ for 5 min, and then washed with MTB for 5 min. The number of adherent platelets was counted in 10 randomly selected fields of 0.25 mm^2^ and at randomly selected time points.

### 4.12. Statistical Analysis

The experimental data were expressed as means ± SEM. Each experiment was carried out at least three times. Statistical analysis for multiple group comparisons were performed by one-way analysis of variance (ANOVA), followed by *post-hoc* Dunnett’s test. A *p*-value of less than 0.05 was considered statistically significant.

## 5. Conclusions

In the current study, the data show that DOX induces mitochondria-mediated intrinsic apoptosis and GPIbα shedding. Dox does not incur platelet activation, however, it obviously impair platelet aggregation and adhesion. Meanwhile, DOX induces mitochondrial ROS generation, and mitochondria-targeted ROS scavenger obviously reduces DOX-induced platelet apoptosis and GPIbα shedding. Thus, mitochondria-targeted ROS scavenger would have potential clinical utility in platelet-associated disorders involving mitochondrial oxidative damage. 
